# Perceptions of Individuals/Patients with Temporomandibular Disorders About Their Diagnosis, Information Seeking and Treatment Expectations: A Comparative Qualitative Study of Brazilian and Spanish Individuals

**DOI:** 10.3390/healthcare14020227

**Published:** 2026-01-16

**Authors:** Luana Maria Ramos Mendes, María Palacios-Ceña, Domingo Palacios-Ceña, María-Luz Cuadrado, Farzin Falahat, Miguel Alonso-Juarranz, Jene Carolina Silva Marçal, Milena Dietrich Deitos Rosa, Débora Bevilaqua-Grossi, Lidiane Lima Florencio

**Affiliations:** 1 Department of Health Sciences, Ribeirão Preto Medical School, University of São Paulo, Ribeirão Preto 14049-900, SP, Brazil; luanamrmendes@usp.br (L.M.R.M.); jene.marcal@usp.br (J.C.S.M.); milenadietrich@alumni.usp.br (M.D.D.R.); deborabg@fmrp.usp.br (D.B.-G.); 2 Grupo de Investigación de Alto Rendimiento en Humanidades e Investigación Cualitativa en Ciencias de la Salud, Departament of Physiotherapy, Ocupational Therapy, Rehabilitation and Physical Medicine, Universidad Rey Juan Carlos, 28922 Madrid, Spain; domingo.palacios@urjc.es; 3 Headache Unit, Department of Neurology, Hospital Clínico San Carlos, 28040 Madrid, Spain; mlcuadrado@med.ucm.es; 4 School of Medicine, Universidad Complutense, 28040 Madrid, Spain; ffalahat@ucm.es; 5 Oral and Maxillofacial Surgery Department, Hospital Clínico San Carlos, 28040 Madrid, Spain; miguelalonsojuarranz@gmail.com; 6 Grupo de Investigación de Alto Rendimiento en Evaluación Multidimensional y Tratamiento del Dolor Crónico, Departament of Physiotherapy, Ocupational Therapy, Rehabilitation and Physical Medicine, Universidad Rey Juan Carlos, 28922 Madrid, Spain; lidiane.florencio@urjc.es

**Keywords:** temporomandibular joint disorders, patient experience, health care service, qualitative research

## Abstract

**Background:** Considering the significant impact on quality of life and the chronic nature of temporomandibular dysfunction (TMD), seeking healthcare is also part of the reality of individuals with this disorder. However, cultural differences and similarities in the experiences of individuals with TMD have not yet been investigated. This study aimed to describe and compare the experiences, beliefs, and sociocultural factors of Brazilian and Spanish individuals with TMD, focusing on their perceptions of the disorder, diagnostic pathways, information-seeking behaviors, and treatment expectations. **Methods:** A descriptive qualitative study was conducted. A purposive sample of 50 participants (25 Brazilian, 25 Spanish), aged 18–50 and diagnosed with TMD according to DC/TMD criteria, was recruited. Data were obtained through semi-structured interviews and analyzed using thematic analysis. **Results:** Six themes emerged, revealing both similarities and differences between the groups. Brazilian participants reported uncertainty about which professional to consult and difficulty accessing specialized care. In contrast, Spanish participants frequently sought physical therapists as their first option and identified them as primary sources of information. Beliefs about TMD etiology varied across samples. Treatment expectations also differed. Brazilians emphasized the difficulty of obtaining effective care, while Spanish participants perceived physiotherapy as being limited to muscular disorders. Perceptions of occlusal splint effectiveness showed variation between the groups. **Conclusions:** These findings underscore the necessity of culturally sensitive approaches to patient care that address not only clinical aspects, but also the sociocultural context that influences health behaviors.

## 1. Introduction

Temporomandibular disorder (TMD) is a term used to describe a group of musculoskeletal and neuromuscular conditions affecting the temporomandibular joint, masticatory muscles, and related structures. These disorders are the leading cause of non-dental pain in the orofacial region [1].

The prevalence of TMD in the global population ranges around 31% in adults. The highest prevalence of the disorder is observed in young and middle-aged adults between 20 and 40 years of age [2]. Moreover, a progressive increase in global TMD prevalence is expected and it may reach 44% in 2050 according to recent estimations [3]. In Brazil, around 39% of the general population has at least one sign or symptom of TMD [4], and these signs and symptoms are more common in women than in men [5]. In the Spanish population, these symptoms are present in 12.5% of adults between 35 and 44 years of age [6]. In a recent meta-analysis, geographic location influenced the prevalence of the disorder, which was higher in South America (47%) than in Europe (29%), and this difference may be related to socioeconomic and psychosocial factors [5]. For example, Brazil faces pronounced socioeconomic inequality and limited access to specialized healthcare services, both of which can delay diagnosis and treatment. Moreover, greater exposure to chronic stressors, such as economic instability and social vulnerability, may increase susceptibility to conditions linked to psychosocial strain. Conversely, Spain benefits from a more robust healthcare infrastructure and stronger social support systems, which can mitigate these risks [7].

Systemic, metabolic, structural, traumatic, psychological, social, and behavioral components have been identified as possible predisposing, causal, and maintaining factors for TMD, suggesting that the disorder has a multifactorial origin [8]. Although cultural factors have been little researched, they are known to influence pain response in this population [9].

Patients with TMD often present craniofacial disability [10] with physical and functional limitations of the orofacial system and psychological components, which have a significant negative impact, affecting activities of daily living and even work, leading to limitations in quality of life [11]. The impact of this disability has already been explored quantitatively in some studies [12,13]. Qualitative studies that have explored the experience of living with TMD highlight the profound effects it can have on work, family, financial, and social lives, and confirm the psychological and mental challenges encountered [14].

Given the significant impact on quality of life and the chronic nature of the condition, seeking health care is also part of these individuals’ reality. A recent meta-synthesis addressed the health care-seeking experience of individuals with TMD. It highlighted the growing need to improve diagnostic pathways and implement patient-centered care for the management of TMD [15]. It was also observed that the effects of the disorder range from mild to profoundly disabling, which can lead to feelings of anxiety, depression, and/or melancholy in patients. These effects are exacerbated by difficulties in accessing medical care, especially at the primary care level [16]. Moreover, the propensity to seek professional assistance appears to be strongly influenced by individual characteristics; patients who pursue treatment more actively often exhibit tendencies toward pain catastrophizing, higher degrees of disability, or severe pain conditions that render self-management approaches insufficient [16].

There are few qualitative studies in Brazil that address the experiences of this population. What is already known is that their experiences include distress until diagnosis, negative psychological aspects such as sadness and suffering, and poor quality of life [17]. In addition, perceptions of associated symptoms and their additional impact, beliefs that could perpetuate these symptoms, and self-management strategies of this population were presented [18]. However, none of the studies focused on the search for diagnosis, information and previous experiences or expectations with treatment. In Spain, qualitative data have not yet been explored in this population and remain an unanswered question. Furthermore, cultural differences and similarities in the experiences of these individuals have not yet been investigated. In other painful conditions, such as chronic low back pain, sociocultural factors have already been described. A qualitative study of the experiences of Brazilians and Spanish people with chronic low back pain showed that Brazilian participants described differences in access to healthcare according to economic status, whereas Spanish participants viewed their healthcare system as sufficiently universal [19].

These two countries share certain sociocultural characteristics, such as historical and linguistic ties. Moreover, the healthcare systems of both countries are founded on the principle of universal and public services, with a similar level of healthcare provision [7] but they differ significantly in access to services, as previously demonstrated in studies on low back pain [17]. However, a comparative analysis of these systems in the context of TMD has not yet been conducted, despite its clear relevance.

Therefore, the objective of this study was to describe and contrast the experiences, beliefs, and sociocultural factors of Brazilian and Spanish individuals with TMD, focusing on their perceptions and understanding of the disorder, as well as their search for diagnosis, information-seeking behaviors, and treatment expectations.

## 2. Materials and Methods

### 2.1. Design

An exploratory and descriptive qualitative study based on a constructivist framework [20] focusing on the experiences of Brazilian and Spanish individuals with TMD was conducted. In this type of design, participants share their experiences to explain everyday events, enabling a detailed understanding of the phenomenon under investigation [21,22,23]. From a constructivist perspective, people interpret the world through the meanings they assign to objects and events within specific contexts. The goal is to explore how individuals respond to diseases or treatments at a given time and in a defined environment [20]. The study adhered to the Consolidated Criteria for Reporting Qualitative Research [24] and the Standards for Reporting Qualitative Research [25].

### 2.2. Ethics

This study was approved by the ethics committee of the Hospital das Clínicas da Faculdade de Medicina de Ribeirão Preto (CAAE 67213923.0.00005440 on 26 April 2023) and the ethics committee of the Hospital Clínico San Carlos (25/097-E on 21 February 2025). Both committees and the current study protocol adhere to the Declaration of Helsinki statement. Participants who agreed to participate signed the informed consent form, which contained all the information related to the study, before the interview.

### 2.3. Participants

Participants were recruited through an intentional, non-probabilistic sample [26]. The total sample consisted of 25 Brazilian and 25 Spanish participants diagnosed with pain-related TMD according to the Brazilian version of the Diagnostic Criteria for TMD (DC/TMD) [27], aged between 18 and 50, of both sexes. This age range criterion was adopted to avoid the inclusion of degenerative processes among TMD diagnoses as we did not have access to imaging exams to confirm or to exclude them. The sample excluded edentulous individuals who were not wearing a prosthesis (given the infeasibility of performing the DC/TMD physical examination), those with a history of systemic diseases (rheumatoid arthritis, fibromyalgia, etc.), neurological disorders (trigeminal neuralgia), a history of head or neck trauma or surgery < 1 year previously, those who had received orofacial treatment in the last 6 months, and those who were unable to cooperate.

Estimating sample size in qualitative research can be challenging, as there are no standardized formulas for calculation [26,28]. Various approaches have been proposed [26,28] and in this study, we used empirical criteria [29,30], and specifically adopted the saturation proposal suggested by Hennink & Kaiser [30]. A saturation proposal by these authors [30] describes that data saturation is achieved between 9 and 17 interviews (with a maximum of 25 interviews) in qualitative studies where the objectives of the study are narrowly defined, the participant groups are homogeneous (participants had information about the phenomenon under study), and interviews are used for data collection. With this proposal, a greater capacity to identify codes, categories, and topics is achieved. In addition, the current proposal helps researchers to determine a reference number to stop collecting data and/or recruiting participants. Finally, the researchers decided to reach the maximum number in both groups to ensure saturation of the information obtained. Conducting approximately 25 interviews is a common approach when aiming for a deeper understanding of the data.

### 2.4. Researcher’s Backgrounds

The research team consisted of ten members (7 women and 3 men), four from Brazil (LMRM, JCSM, MDDR, and DBG) and six from Spain (MPC, DPC, MLC, FFN, MAJ, and LLF). Six of the researchers are professors and doctors (MPC, DPC, MLC, FFN, DBG, and LLF), one is a physician, one is a PhD student (LMRM), and two are master’s students (JCSM and MDDR). Six of the team members are physiotherapists, three are physicians and one is a nurse. The researchers have experience or training in qualitative methodology, as well as experience in the fields of chronic pain and temporomandibular disorders. The researchers responsible for applying and confirming the inclusion criteria (JCSM, LMRM) for potential participants had no clinical and/or therapeutic relationship with them.

Before the start of the study, DPC, a professional with extensive experience in qualitative research, guided the other researchers through comprehensive training on all phases of study planning, including conducting pilot interviews.

### 2.5. Data Collection

Participants were recruited without any prior contact with the researchers. All individuals underwent an in-person TMD diagnosis based on the DC/TMD criteria. Those who agreed to participate completed a form to provide sociodemographic data, details about TMD and related comorbidities such as headache and neck pain and answered the Craniofacial Pain and Disability Inventory (CF-PDI) questionnaire to characterize the sample. The CF-PDI is a multidimensional tool that assesses psychosocial factors, pain, and disability related to the orofacial region. It consists of 21 items with a four-point ordinal scale for each item. The maximum score is 63, and the higher the score, the greater the TMD-related disability. CF-PDI data will be presented only as total scores since the Spanish version (original) has two dimensions and the Brazilian version has three [31]. In Brazil, the in-person assessments were conducted at the Laboratory for the Analysis of Human Posture and Movement at the Ribeirão Preto Medical School, University of São Paulo, Ribeirão Preto-SP. In Spain, the in-person assessments were conducted at Hospital Clínico San Carlos, Madrid, Spain.

**Figure 1:**
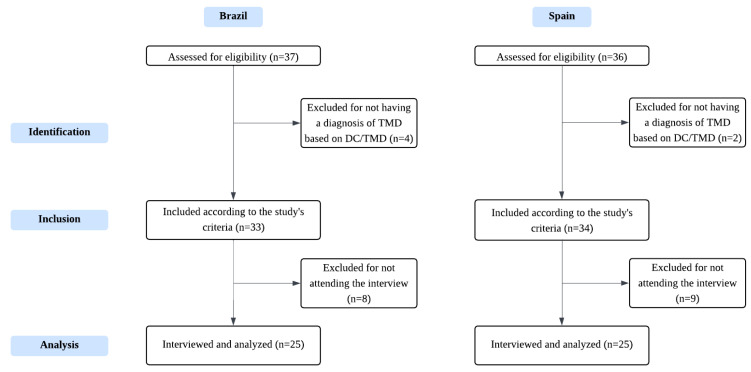
Data collection flowchart. DC/TMD: Diagnostic Criteria for Temporomandibular Disorders.

After confirming eligibility, participants took part in a single in-depth interview lasting approximately 30 to 60 min (Figure 1). Interviews with Brazilian participants were conducted by a physical therapist and a master’s student (JCSM), while those with Spanish participants were conducted by a physical therapist and a PhD student (LMRM). The interview followed a guide with open and semi-structured questions designed to explore areas of interest related to the phenomenon under investigation. The development of this guide prioritized content and response process validity [32], beginning with a literature review aligned with the study’s main objective [12,13,14]. After its initial construction, the research team held sessions to identify missing dimensions, assess question clarity, and refine wording. Adjustments were made to improve flow and incorporate additional relevant content. Key topics discussed included their perceptions of the disorder, diagnostic pathways, information-seeking behaviors, and treatment expectations [32].

### 2.6. Data Analysis

All interviews were transcribed for textual analysis, and each participant was assigned a code to ensure anonymity during data processing. The Brazilian participants were coded as BP and the Spanish participants as SP. The data were analyzed using an inductive approach [21,26,29,33]. A codebook-based thematic analysis [34,35,36] was conducted for both samples. The process began by identifying relevant excerpts from the transcripts that addressed the research objectives [33]. These excerpts were then coded to represent descriptive content, and the codes were grouped into categories based on similarity. Finally, the categories were reviewed and organized into overarching themes.

A collaborative coding approach was used for all interviews [37]. Two physical therapists and master’s students (JCSM and MDDR) jointly coded the Brazilian sample. One researcher analyzed the first 13 interviews, while the other coded the remaining 12, identifying initial codes and potential category groupings. For the Spanish sample, coding was performed by a physiotherapist and doctoral student (LMRM) and a physical therapist with a PhD (MPC). The first coded all even-numbered interviews, and the second coded all odd-numbered ones. Subsequently, these researchers, together with a third team member (LLF), convened to review and consolidate the codes and finalize the categories for both samples [38].

Next, one researcher (LMRM) compared codes and categories across samples, highlighting similarities and differences. Additional team meetings were held to integrate findings, review data collection and analysis procedures, and ensure triangulation. During these sessions, final themes were presented, refined, and derived from the categories. Discrepancies were resolved through consensus among the research team. The results were then organized to illustrate similarities and differences between samples, supplemented with narrative examples for contextualization. A thematic analysis was conducted using MAXQDA software (version 2024-Release 24.11.0, VERBI GmbH, Berlin, Germany).

**Figure 2:**
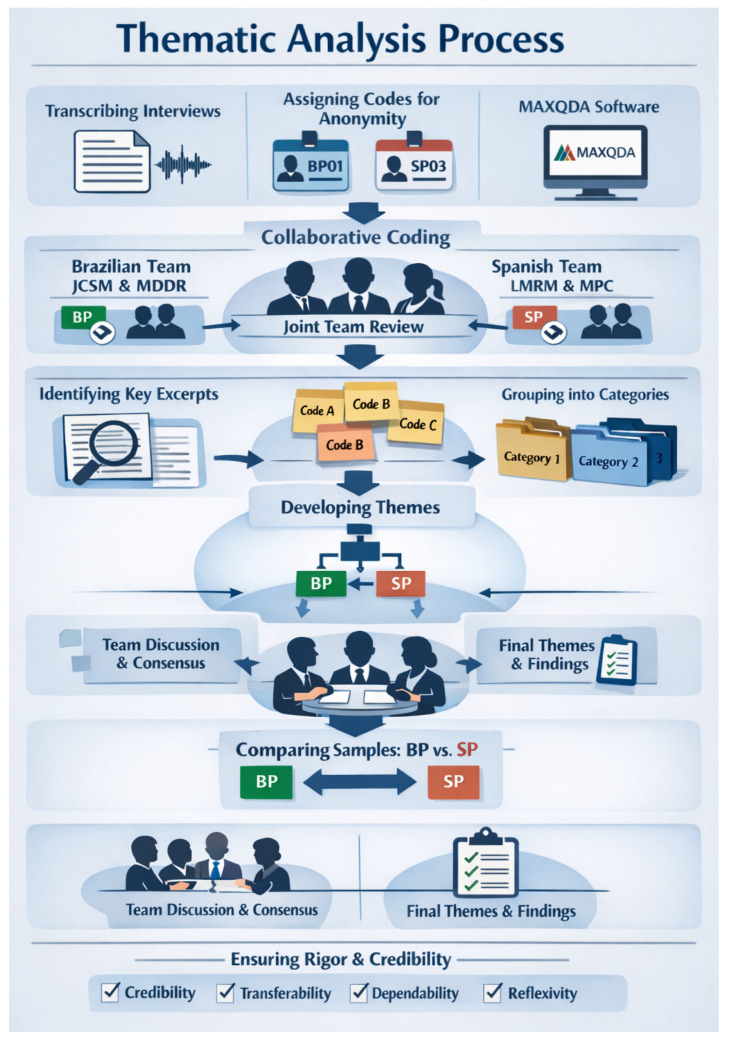
Flowchart of the Thematic Analysis Process (generated using artificial intelligence). BP: Brazilian participant; SP: Spanish participant; BP01 and SP03 are examples of codes assigned to each participant in their respective countries to ensure anonymity while maintaining identification of the country of origin during the analysis and reporting process.

To ensure reliability and methodological rigor, the criteria of credibility, transferability, dependability, and reflexivity were applied [29,39] (Figure 2). Participants did not provide feedback on the findings.

## 3. Results

Fifty patients were included in the study. Of these, 25 participants were Brazilian (88.00% women), with an average age of 29 years (SD = 6.45), a median disorder duration of 6.5 years (IQR = 6.25), and a predominance of mixed TMD diagnoses (56.00%). The Spanish sample consisted of 25 participants (80.00% women), with an average age of 33 years (SD = 8.47), a median disorder duration of 4 years (IQR = 6.00), and a predominance of mixed TMD diagnoses (80.00%). Table 1 presents the sociodemographic and clinical details of the 50 patients.

After conducting a thematic analysis, 4690 codes were identified in the Brazilian interviews and 4645 in the Spanish interviews. Six main themes emerged from these codes: the search for diagnosis and specialized care; sources of information about the disease; perceptions and beliefs about the causes of TMD and the influence of emotions on symptoms; previous experiences and expectations of good treatment; physical therapy treatment; strategies for dealing with TMD.

### 3.1. The Search for Diagnosis and Specialized Care

In this context, the narratives from both samples are similar. Participants repeatedly mentioned the difficulty or absence of diagnosis for their symptoms. Some reported receiving a diagnosis from a physician or dentist, but most were diagnosed only with bruxism, not TMD.

*“I went to see a friend of mine who is a dentist, and she told me that I was losing teeth, right? And that it could be bruxism.” BP05*

*“So I went to the doctor, the general practitioner, and he told me that what I had was bruxism.” SP36*

In most cases, people sought health care after experiencing numerous episodes of pain, difficulty performing everyday tasks, and discomfort caused by joint noises. The desire to find a solution was also a main motivator. Physicians and dentists were the professionals most frequently mentioned for the initial consultation. Initially, these professionals were not TMD specialists but ultimately referred patients to one.

*“I don’t remember exactly why I went to look for it, but I remember that I was in a lot of pain and that I felt more cracking at that moment.” BP02*

*“Because it’s true that it was really uncomfortable for me, so I went to the doctor.” SP08*

The Spanish sample mentioned cases of misdiagnosis. The importance of having an accurate diagnosis was also discussed. This means giving the symptom a name so it can be managed in the best possible way. Brazilian participants highlighted one difficulty they faced in seeking health care: finding a professional who specializes in TMD. This was due to a lack of knowledge about which professional to see and where to find them. Additionally, the high cost of consultations was considered an impediment to following up on the condition once they did find a specialist.

*“And maybe I wouldn’t have this bruxism that I have now. If I had been diagnosed correctly from the beginning.” SP23*

*“I would be glad to have it (the diagnosis) so that I can take action as soon as possible.” SP19*

*“I don’t know if I should go to the doctor, I don’t know if I should… I don’t know exactly which professional to go to. Whether it’s the doctor, the dentist, the physical therapist, I don’t know.” BP04*

Only Spanish participants stated that although they had to wait several months for specialized care, the referral process was relatively simple. Only two Brazilian participants mentioned the referral process, and both referred to referrals in the private system without mentioning any facilitators or barriers during this process. Another notable aspect was that Spanish participants preferred physical therapy as their initial treatment.

*“I’ve had it very easy because my primary care physician is actually a very diligent person.” SP31*

*“At first, I went to the physical therapist because the pain was, well, although it was uncomfortable, it was tolerable.” SP28*

### 3.2. Sources of Information About the Disease

Participants in both samples reported using the internet as an information source. They relied on search engines and artificial intelligence tools to find explanations for their symptoms and possible treatments. Additionally, they watched videos to learn strategies and perform exercises for symptom management and used social media to search for specialized professionals. Many participants mentioned seeking information from healthcare professionals, considering them the most suitable source. Specifically, doctors and dentists were mentioned. Some participants also stated that their training in health helped them find information.

*“It was the times I sought help, like dentists, doctors, and then I read about (uhum) I read about and… I think that by talking like this, you discover that many people have discomfort.” BP09*

*“Well, it has always been through doctors or dentists.” SP35*

The Spanish sample also included participants who did not fully trust information found on the internet. Additionally, the importance of physical therapists in understanding this disease was emphasized. Some even mentioned the need for speech therapists to support the treatment of the disorder.

*“There are so many things on the internet that you must take with a grain of salt. So, well, you read a lot of things. Some you believe, others you don’t. Well… Useful… Well, just information, no, you can’t believe it 100%.” SP29*

*“I looked online, but above all, I looked for people who could help me. That means physical therapists and even speech therapists.” SP32*

### 3.3. Perceptions and Beliefs About the Causes of TMD and the Influence of Emotions on Symptoms

Participants’ beliefs about the causes of the disorder in both countries were similar. They reported that pain onset was associated with stress and concerns relating to life changes and increased responsibilities at certain stages (e.g., undergraduate or postgraduate studies, job or country relocation), as well as financial and personal concerns. Habits such as teeth grinding and sleep disturbances were also mentioned. Some people could not identify the cause of the pain.

*“There is a very important factor in my life that was very significant, and that is my concern about leaving elementary school, where I didn’t have so many responsibilities and so much…” BP15*

*“It started when I began my master’s degree.” SP28*

*“When the clenching is more intense, it intensifies the headache and intensifies the pain in the face itself, and the clenching is like this every day.” BP21*

*“It must be that he had been clenching his jaw or whatever for years.” SP18*

Meanwhile, some participants in the Spanish sample reported that the onset of facial pain was related to episodes of subluxation and the completion of orthodontic treatment.

*“About 22 years ago, more or less, we were at the amusement park, and I wanted to eat that, and that’s when I got dislocated.” SP02*

*“And the truth is that since then (orthodontic treatment), I have been in much more pain, and my quality of life has worsened.” SP23*

In both samples, some participants reported being unable to perceive the influence of emotions on their symptoms, either because they were unaware of them at the time or because they could not identify a direct relationship between pain and emotions. In contrast, many said they had noticed an intrinsic relationship between stress and the onset or exacerbation of symptoms, considering stress to be the main influence on pain. Stress was also associated with increased teeth clenching and muscle tension, which can trigger facial pain. Additionally, emotions such as nervousness, worry, anxiety, feeling overwhelmed, and sadness were mentioned as being related to symptom variation.

*“When I’m overwhelmed with college too, when I have a lot of assignments to hand in and a lot of things to do, I also feel a lot more pain, both in my face and in my head.” BP28*

*“So, if I’m more stressed or angry, will I clench my teeth more, and will that aggravate the joint even more?” SP20*

### 3.4. Previous Experiences and Expectations of Good Treatment

In this context, the narratives from both samples are similar. The search for specialized professionals or specific treatments, such as physical therapy, acupuncture, and botulinum toxin (Botox) application, was highlighted, in addition to the use of medication prescribed by physicians and bite splints recommended by dentists. Most reports on therapeutic approaches in the Brazilian sample indicate that participants had not yet tried them. This was due to several factors, including the difficulty of obtaining a diagnosis and finding a specialized professional.

*“I was in my hometown and couldn’t find a physical therapist. I wanted to see one, but there was no one available, and I couldn’t find anyone to contact. So, in reality, I never received treatment, no, I never did.” BP19*

Patients have several expectations regarding effective treatment. They want to seek information to understand the causes, receive an accurate diagnosis, and become familiar with the treatment process. Patients also want to prevent pain from starting and participate more actively in treatment by learning pain self-management strategies. They expect professionals to listen to them and acknowledge the reality of their problem. Treatment is expected to reduce or eliminate pain, clicking, and tension by addressing the underlying cause. Additionally, treatment is expected to prevent major problems in the future.

*“Good treatment… I think the professional should sit down and explain what is happening, why I am doing that.” BP01*

*“For me, a good diagnosis that identifies the exact cause of why.” SP33*

*“Then whoever listens to you, whoever notices that we’ve already done this, that you’re doing this, that you’re doing that… Then whoever pays attention to you.” SP02*

In the Spanish sample, some participants mentioned, in relation to their expectations, the importance of having a specialized and individualized protocol, as well as effective treatments that avoid unnecessary expenses. They also expressed a desire to avoid invasive procedures and considered that a multidisciplinary approach would be more effective.

*“And that there should also be a protocol in place so that we can act relatively quickly, that is, define what is happening, take action, and refer cases as needed, without it taking too long.” SP32*

### 3.5. Physical Therapy Treatment

Knowledge about physical therapy as a treatment option varied among participants in both samples. Some had never heard of it, while others were familiar with its application in this area through recommendations from other health professionals or previous treatment experiences. Participants also mentioned the difficulty of finding a specialist in this area.

*“I know that physical therapy… physical therapy includes TMD. I know people who work in that field, who are physical therapists specializing in that, so I see those people quite often.” BP08*

*“But it is certainly the most effective. For me, it has been the most beneficial so far. Without a doubt, it is the one I would choose for treatment.” SP14*

Regarding the role of physical therapy, they stated their expectation that pain and associated symptoms would be reduced through manual therapy techniques and exercises. They also noted that, while physical therapy would not make the problem disappear, it would help with self-management and symptom control.

*“Get well, be able to eat, have no more headaches after treatment, sleep better, of course.” BP06*

*“I’m going to the physical therapist to relieve the entire area.” SP16*

Some Spanish participants stated that physical therapy is only important for cases of muscular disorders because it can relax and reduce tension. However, they did not consider physical therapy to be useful for joint disorders. These Spanish participants also mentioned treatment costs as an impediment to undergoing treatment more frequently or even starting it in the first place.

*“Or I imagine that if you go there now and you’re not muscular and you’re articular, I don’t think it will help much.” SP19*

*“The problem with physical therapy and these types of therapies, as well as dentists and things like that, is that they are very expensive.” SP14*

### 3.6. Strategies for Dealing with TMD

The strategies used to control facial pain were diverse in both samples. Some participants stated that they did not have any strategies, either because they did not know the cause of the pain or a specific solution, or because they forgot about it when it was not bothering them. However, most reported self-management strategies that included relaxation and stress management techniques, rest, massage, exercise, jaw movements such as opening and closing the mouth to relax the joint, limiting movement, applying hot and/or cold compresses, using anti-inflammatory cream and avoiding ‘apartment’ (placing the tongue on the palate and keeping the mouth open). The use of an occlusal splint was also cited as a strategy.

*“Massage: at bath time, giving a little relaxing massage helps a lot.” PB33*

*“Well, sometimes I apply heat myself or give myself a mini massage here.” SP16*

The Brazilian sample mentioned changing habits and routines as effective strategies for relieving facial pain, whether by improving sleep quality or addressing emotional factors. They also cited chewing gum as a strategy. A participant from the Spanish sample pointed out the use of the oral screen for treating facial pain.

*“I’ve tried to change some things in my routine, catch up on my exercise, but honestly, I’m not doing as well as I should.” BP02*

*“I clench my teeth, and I have a habit of chewing gum because it helps me improve.” BP27*

*“I use an orofacial screen. I started doing this recently because it was also recommended to me to work on my muscles.” SP32*

Experiences with the occlusal splint varied among the Spanish sample: some participants reported considerable improvement and considered it essential, using it consistently; others, however, could not tolerate it and experienced worsening symptoms when using it, requiring additional treatment. The Brazilian sample considered it an effective strategy.

*“I sleep with the occlusal splint, right? Every day. If I don’t use the occlusal splint, I have a headache, pain… here (points to the right TMJ) all day long, like this.” BP24*

*“And an occlusal splint… It’s true that I don’t tolerate it very well because I still clench my teeth even when I’m wearing the occlusal splint.” SP16*

## 4. Discussion

This study compares experiences, beliefs, and sociocultural factors of Brazilian and Spanish individuals with TMD, focusing on their perceptions of the disorder, diagnostic pathways, information-seeking behaviors, and treatment expectations. Through this comparison, we can highlight differences and similarities between the two groups and the influence of sociocultural factors on the TMD experience. These differences and similarities were evident in participants’ approaches to seeking diagnosis and specialized care. In the Brazilian sample, a lack of knowledge about which healthcare professional to consult was commonly reported, whereas participants in the Spanish sample more frequently sought physical therapists, who were also cited as important sources of information. Spanish participants often associated the disorder with orthodontic treatment and episodes of joint locking. In contrast, Brazilian participants emphasized difficulties in accessing specialized care. Additionally, Spanish participants perceived physical therapy as effective only for muscular disorders. Finally, a clear difference emerged in perceptions regarding the use and effectiveness of occlusal splints in managing the disorder.

Regarding the search for diagnosis and specialized care, Brazilian participants reported uncertainty about whom to consult, whereas Spanish participants identified physical therapists as their first option. Previous studies have also noted this difficulty in determining the appropriate professional [40,41], a factor that may contribute to delays in accessing effective treatment [41]. The search for physical therapists as the first choice may be related to these participants’ prior knowledge of the benefits of physical therapy, either for this disorder or because they have undergone previous treatments. This has also been pointed out in previous studies [42].

Continuing the discussion on seeking diagnoses and specialized care, it is important to highlight the differences between the two countries’ health systems. Spanish participants reported that obtaining a referral to a specialist within the public health system was relatively easy and quick, whereas Brazilian participants did comment on the referral process in the public system. Although the health systems of both countries are founded on the principle of universal, free healthcare, they differ markedly in their organization and socioeconomic contexts. Spain’s health system underwent major organizational reforms between the 1970s and 1980s, resulting in a structured model with access through primary care teams (family physicians) and an established referral pathway to specialized services. Brazil’s system is similarly grounded in universal access; however, as a more recent system operating within a developing-country context, it faces socioeconomic challenges that affect its effective implementation. In the specific context of orofacial pain, Spain has a well-established referral system to specialists, such as maxillofacial surgeons, whereas such referral pathways are less clearly defined in Brazil [7]. In other words, participants from both countries have different experiences with the healthcare system, particularly regarding referrals to specialized services. Previous studies have indicated that long waiting times for appointments or referrals can worsen symptoms, lead to incomplete treatment, prolong recovery, and increase worries [16,41,43,44]. These differences reflect underlying systemic features, such as the organization of referral networks and the distribution of resources, that directly shape patient trajectories and perceived quality of care.

Another noteworthy point is that both groups of participants reported bruxism as their sole formal diagnosis received from health professionals. Bruxism, encompassing a range of muscular activities, is often considered a gateway to dental care [45]. Although it is not a definitive diagnosis, it frequently acts as a trigger for patients to seek treatment. Moreover, bruxism has been suggested as a potential contributor to TMD-related pain, as patients with TMD tend to exhibit a higher frequency of awake bruxism compared to healthy individuals. In addition, it is hypothesized that additional factors (such as psychological stress, inadequate sleep, comorbidities, and other non-painful musculoskeletal symptoms) may play significant roles in the development and persistence of both TMD and bruxism [46]. This demonstrates the intrinsic relationship between the two conditions.

The sources of information for both samples were the same—namely, the internet and health professionals—as highlighted in previous studies [47,48]. Both samples identified physicians and dentists as sources of information about the disorder. However, Spanish participants also sought information from physical therapists. This preference appears to be related to the fact that some participants were undergoing or had previously undergone physical therapy for unrelated health conditions. A prior study supports this observation, showing that individuals often take advantage of consultations for other complaints to seek advice or explore treatments perceived as low-risk and low-burden. This behavior may be intentional—when participants actively seek guidance during appointments—or incidental, when the health professional spontaneously introduces the topic and suggests treatment options [42]. This may highlight greater access to or demand for physical therapy treatment by Spanish participants.

There were also notable differences in perceptions and beliefs regarding the causes of TMD. Spanish participants often attribute the onset of their facial pain to orthodontic treatment or episodes of joint dislocation. While TMD is recognized as a multifactorial condition influenced by both physical and psychosocial factors [8], current evidence indicates that orthodontic treatment neither increases nor decreases the risk of TMD [49]. Therefore, these findings reveal a misconception among participants, highlighting the need for accurate information to guide patient understanding and decision-making.

Regarding previous experiences and expectations for effective treatment, Brazilian participants reported difficulties in obtaining a diagnosis and accessing specialized professionals. Similar findings have been documented in other studies [14,16], in which the absence of a clear diagnosis led some individuals to question the legitimacy of their symptoms and repeatedly seek healthcare services. This emphasizes the critical role of diagnosis, as it not only validates patients’ experiences but also influences their ability to pursue appropriate treatment. In some cases, participants expressed greater concern about receiving a diagnosis than about the treatment options themselves [44]. Furthermore, delays in diagnosis have been described as frustrating and anxiety-provoking, often accompanied by fear that the pain could indicate a more serious condition [40,44,50]. This diagnostic uncertainty also contributed to self-doubt, hindered progress, and prompted individuals to develop their own explanations for their symptoms [40,43,47,50].

Regarding physical therapy treatment, Spanish participants expressed the belief that it was only effective for muscular disorders. This perception was also observed in the study by Dinsdale et al. (2025) [42], where participants reported that physical therapy could alleviate symptoms such as muscle tension and weakness. However, when faced with more serious conditions—particularly those involving the temporomandibular disc or joint—many believed that a more invasive intervention would be necessary [42].

The coping strategies reported by both groups were similar and aligned with findings from previous studies. However, perceptions regarding the use of occlusal splints differed: Brazilian participants generally reported positive outcomes and symptom improvement, whereas Spanish participants more frequently described discomfort associated with their use. Like the participants’ perceptions, evidence on the effectiveness of occlusal splints is mixed. Although they have been shown to have benefits in the treatment of TMD, mainly in pain relief and improved function, the evidence is controversial when they are used as a single treatment without combination with other therapeutic modalities [51]. The difference in perceived comfort when using occlusal splints may be related to factors such as splint type, material, and quality of design [52]; recommended use duration; and health professional monitoring. As these factors are considered relevant for interpreting the results, their absence from the participants’ narratives represents a limitation of our study.

This is the first study to examine and contrast experiences from individuals with TMD from distinct countries and cultures. Understanding how these factors influence the search for diagnosis, access to information, and specialized treatment, as well as beliefs about the causes of TMD, provides valuable insights for improving care pathways. Importantly, cultural differences may also influence long-term prognosis, potentially contributing to chronicity and greater disability, as highlighted in previous research [53]. One of the strengths of these findings is the insight they provide into differences among health systems, particularly regarding access to specialized care. From this perception, strategies can be developed to improve the system using successful approaches. The option to conduct interviews online can be considered a strength of the study, as it provided participants with greater geographical flexibility and allowed them to choose a setting where they felt most comfortable and safe to share their experiences.

One limitation of this study is the predominance of women in the sample, which reflects the most common TMD profile in the general population [2]; however, this also restricts the interpretation of the findings. It is also important to recognize that these findings cannot be generalized, which represent another limitation. Nevertheless, qualitative research does not aim for generalization but seeks to provide an in-depth understanding of specific realities, considering the uniqueness and individuality of human experiences [54]. A comprehensive and detailed description of the context, participants, procedures, and studied phenomena was provided to allow readers to assess the potential transferability of the findings to similar settings [39]. Some of the participants’ information was not registered quantitatively, as the diagnoses formally received from health professionals or their treatment history (previous or current), such as how many of them were using splints or which professionals they were being monitored by at the time of the interview. Future studies may consider extending the information on participant’s treatment to better characterize the sample. Finally, in qualitative studies, various approaches exist for determining sample size, such as saturation [26,28,29,30]. In this study, the researchers applied an empirical saturation approach that allowed them to establish a range and a maximum number of interviews required to achieve saturation [30], provided that the following conditions were met: having clearly defined objectives, homogeneous groups, and using interviews as the data collection instrument. Given that the present study addresses cultural aspects, it might be necessary to apply additional criteria specific to cross-cultural studies. For instance, Hagaman and Wutich [55] in their work on how many interviews are sufficient in multisited and cross-cultural research, reported that 16 interviews or fewer were adequate to identify common themes in sites with relatively homogeneous groups. However, these authors [55] noted that larger samples—between 20 and 40 interviews per site—would be necessary to achieve data saturation for metathemes. We believe that using a sample of 25 participants (with one interview each) in each group (Brazil and Spain) also meets the sample criteria for cross-cultural studies.

## 5. Conclusions

This study highlights the experience of living with TMD under distinct sociocultural contexts in Brazil and Spain. Although both groups employed similar coping strategies, notable discrepancies emerged in their diagnostic pathways, sources of information, and treatment expectations. Brazilian participants reported uncertainty about which professional to consult and difficulty accessing specialized care. In contrast, Spanish participants frequently sought physical therapists as their first option and as a source of information. Beliefs about the causes of TMD and the scope of physical therapy also diverged, with Spanish participants perceiving physical therapy as limited to muscular disorders. Additionally, perceptions of occlusal splint effectiveness varied between the two groups. These findings underscore the necessity of culturally sensitive approaches to patient care that address not only clinical aspects, but also the sociocultural context that influences health behaviors.

## Figures and Tables

**Table 1:** Demographic and clinical characteristics of the sample.

Variables	Brazil (N = 25)	Spain (N = 25)
Age (years)	29.00 (6.45) ^a^	33.64 (8.47) ^a^
BMI (Kg/cm^2^)	23.89 (4.24) ^a^	24.68 (3.82) ^a^
Duration of TMD symptoms (years)	6.50 (6.25) ^b^	4.00 (6.00) ^b^
Mouth opening (without pain) (mm)	43.05 (7.93) ^a^	39.14 (10.77) ^a^
Mouth opening (with pain) (mm)	51.91 (6.48) ^a^	50.04 (7.54) ^a^
Number of tender points (right)	5.92 (2.74) ^a^	7.48 (3.42) ^a^
Number of tender points (left)	4.88 (3.22) ^a^	6.96 (3.74) ^a^
CF-PDI score	19.00 (14.00) ^b^	20.00 (16.00) ^b^
Sex		
Female	22 (88.00%)	20 (80.00%)
Male	3 (12.00%)	5 (20.00%)
TMD diagnosis		
Pain-related	10 (40.00%)	5 (20.00%)
Intra-articular	1 (4.00%)	0 (0%)
Mixed	14 (56.00%)	20 (80.00%)
Marital status		
Single	14 (56.00%)	19 (76.00%)
Married	11 (44.00%)	5 (20.00%)
Divorced	0 (0.00%)	1 (4.00%)
Education		
Basic Level	0 (0%)	0 (0%)
Secondary Level	3 (12.00%)	4 (16.00%)
Professional Level	0 (0%)	2 (8.00%)
Incomplete Graduate Level	7 (28.00%)	1 (4.00%)
Complete Graduate Level	5 (20.00%)	15 (60.00%)
Postgraduate Level	10 (40.00%)	3 (12.00%)
Occupation		
Employed	19 (76.00%)	24 (96.00%)
Unemployed	0 (0%)	0 (0%)
Student	6 (24.00%)	1 (4.00%)
Jaw Joint noise		
Yes	25 (100.00%)	22 (88.00%)
No	0 (0%)	3 (12.00%)
Headache		
Yes	22 (88.00%)	24 (96.00%)
No	3 (12.00%)	1 (4.00%)
Neck Pain		
Yes	20 (80.00%)	19 (76.00%)
No	5 (20.00%)	6 (24.00%)
Tinnitus		
Yes	13 (52.00%)	12 (48.00%)
No	12 (48.00%)	13 (52.00%)

BMI: body mass index; CF-PDI: Craniofacial Pain and Disability Inventory; a: data presented as mean and standard deviation; b: data presented as median and interquartile range.

## Data Availability

The data presented in this study are available on request from the corresponding author due to ethical reasons.

## Abbreviations

The following abbreviations are used in this manuscript:

TMD	Temporomandibular disorders
DC/TMD	Diagnostic Criteria for TMD
CF-PDI	Craniofacial Pain and Disability Inventory
